# The Physicians’ Opportunity

**Published:** 1916-09

**Authors:** 


### The Physicians' Opportunity.

The physicians in every city, town and village should avail themselves of the opportunity to bring home some forceful truths to the laity, at this time. The people are in a mood to listen and to act. Just at present all eyes are upon New York and the people are dreadfully worked up over the fact that one thousand two hundred and sixty persons (mostly babies) have died of infantile paralysis since January, 1916.

This is a waste of precious life and a condition to be deplored. It is to be hoped that some treatment will be found that will prove effective.

But why not call the attention of the people now that they are awake and in a listening attitude, to the fact that diphtheria, scarlet fever, typhoid fever, measles and whooping cough are much more dangerous as causes of death than infantile paralysis. Tuberculosis is many times more important than infantile paralysis, both as regards the amount of sickness it causes and the number of people it kills.

The people should be convinced that typhoid fever need not exist at all if all that is known about it were actually utilized.

The bubonic plague of today is identical with the black death of the middle ages. It is estimated that every case of human plague costs the municipality in which it occurs $7500. It is well known to physicians that rats are the carriers of the pest bacillus, which occurs in three forms, viz: the pneumonic, with a death rate of almost 100 per cent; the septicaemic, which is nearly as fatal, and the bubonic, which claims 50 per cent of its victims—but are the people making war on rats? Would they want these pests in the home if they could be made to understand how great the danger? There is absolutely no excuse for the existence of the fly, the mosquito, the rat or mouse, and a number of other things, not mentionable in polite society. If the general public could be awakened and kept awake long enough to become convinced and aroused to these facts what a cleaning up there would be. But there must be "line upon line and precept upon precept," and it is greatly to be feared that until the end of time we shall have these unwelcome, obnoxious pests among us, but the physician who does his whole duty will not fail to call attention to these conditions and to tell the people how they may be overcome.

